# Artificial intelligence in orthopedic research assistance: a resident’s perspective

**DOI:** 10.1007/s12306-025-00894-w

**Published:** 2025-02-17

**Authors:** Rubén Dario Arias Perez, Ricardo Londoño Garcia

**Affiliations:** Pontifical Bolivarian University, Medellín, Colombia

**Keywords:** Artificial intelligence, Large language model, Orthopaedic surgery, Orthopaedics, Research enhancement, Generative AI models

## Abstract

Artificial intelligence (AI) is transforming orthopedic research by optimizing academic workflows, improving evidence synthesis, and expanding access to advanced data analysis tools. Generative AI models such as ChatGPT and GPT-4, alongside specialized platforms such as Consensus and SciSpace, empower researchers to refine search queries, enhance literature reviews, synthesize documents, and conduct advanced statistical analyses. These technologies enable the interpretation of large datasets, saving time and boosting efficiency. For orthopedic residents, AI is particularly impactful, revolutionizing their education and fostering greater independence in research. This review explores the key applications of AI as a research assistant in orthopedics, as well as its ethical considerations and challenges.

## Introduction

Orthopedic research is an important part of improving patient care, including everything from surgical innovations and rehabilitation therapies to the development of novel biomaterials and disease progression prediction models [1, 2]. This field demands rigorous precision, exhaustive literature reviews, and sophisticated statistical analyses to produce findings that can reliably improve clinical practice [3]. However, the exponential growth of medical literature and the increasing complexity of data present significant challenges for researchers. The large volume of information available often causes delays in synthesis and decision-making, highlighting the need for innovative approaches to streamline research workflows [4].

In recent years, artificial intelligence (AI) has emerged as a transformative tool in orthopedic research, offering solutions to overcome these challenges. AI technologies automate repetitive tasks, enable the efficient extraction of insights from vast and diverse data sources, and enhance the synthesis of evidence, fundamentally reshaping how orthopedic studies are conducted [5]. These advancements not only save time but also reduce errors and enhance the quality of analyses, providing researchers with more reliable results in less time [6].

**Fig. 1:**
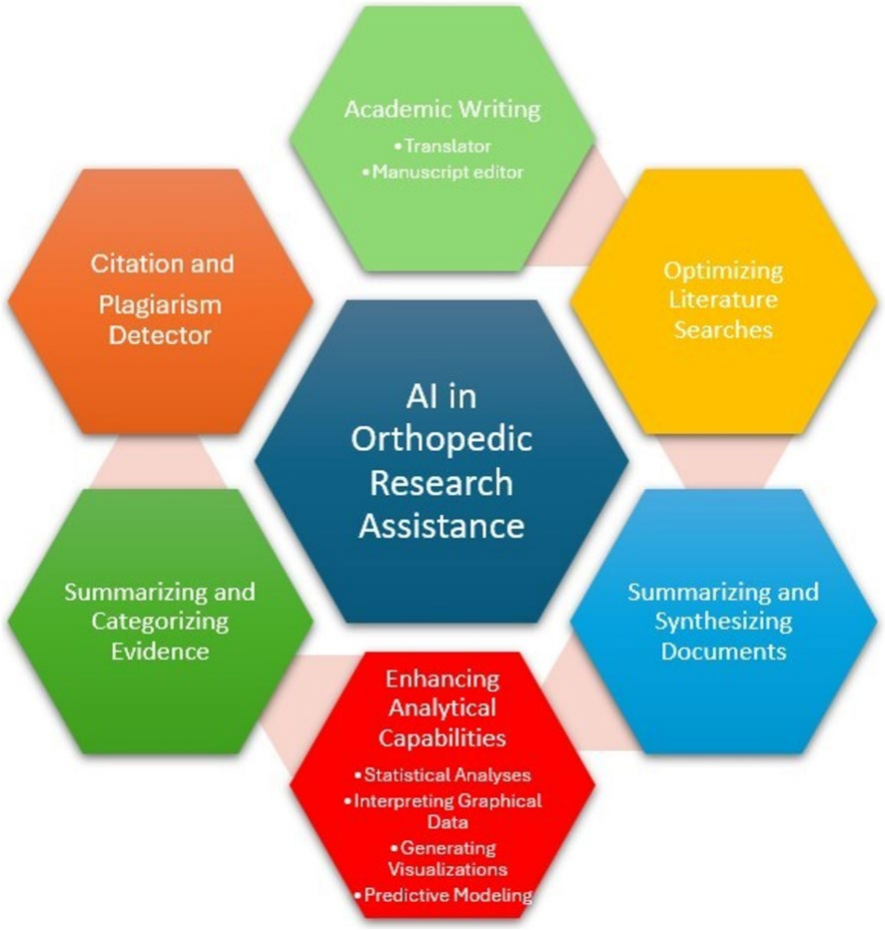
AI in orthopedic research assistance

One of the most notable applications of AI in orthopedic research is through generative AI models, such as ChatGPT, Claude, DeepSeek or Bard [1]. These tools provide intuitive, user-friendly interfaces that assist researchers in drafting, summarizing, and refining manuscripts. They help structure complex ideas into coherent narratives, making the writing process more efficient and accessible, especially for residents and early-career researchers who may still be developing their academic writing skills [4] (Fig. 1).

Additionally, advanced machine learning algorithms are revolutionizing data analysis in orthopedics. These algorithms can process and analyze large datasets, identifying trends and patterns that may not be immediately apparent through traditional methods [2]. For instance, predictive models generated by AI can predict clinical outcomes, identify high-risk patient populations, and assess the long-term efficacy of implants or surgical techniques [1]. This capability enhances the precision of research and contributes to more personalized and effective patient care. AI also personalizes learning by recommending relevant resources and automating repetitive tasks, allowing residents to focus on critical thinking and innovation [2]. This ultimately improves the quality and efficiency of research, contributing to better patient care and advancing the field of orthopedics.

As AI technology advances, its role in orthopedic research will continue to grow, enhancing productivity, precision, and innovation. This review offers a comprehensive overview of AI’s current applications in orthopedics, highlighting its transformative potential while stressing the importance of ethical use. By embracing these technologies thoughtfully, the orthopedic field can overcome existing challenges and unlock new opportunities for improving patient care and research.

## Academic writing: AI in drafting and refining manuscripts

Academic writing is an essential pillar of scientific advancement, allowing researchers to disseminate findings, contribute to collective knowledge, and inspire clinical innovations [7]. For orthopedic researchers and residents, balancing the demands of clinical practice with the precision required for high-quality academic writing can be particularly challenging [8]. Crafting manuscripts that are clear, well-structured, and formatted to the exacting standards of peer-reviewed journals is a time-intensive process that demands significant effort and expertise [7].

AI tools have emerged as transformative allies in this domain, simplifying academic writing and making it more efficient and accessible. Platforms such as ChatGPT, GPT-4, Claude, Bard, Jenni AI, Aithor, and Paperpal automate many of the labor-intensive tasks involved in manuscript preparation (Table 1). These generative AI models excel in producing well-organized drafts based on user-provided inputs, such as study data, research notes, or objectives [7].

**Table 1:** AI tools and functionalities in orthopedic research

Tools	Citation and plagiarism detector	Academic writing	Literature searches	Summarizing and synthesizing documents	Analytical analysis	Summarizing and categorizing evidence
ChatGPT	–	✓	–	✓	✓	✓
Claude	–	✓	✓	✓	–	✓
Aithor	–	✓	–	–	–	–
Paperpal	–	✓	–	–	–	–
Consensus	–	–	✓	✓	✓	✓
Jenni AI	–	✓	–	–	–	–
SciSpace	–	–	✓	✓	–	✓
Grammarly	✓	✓	–	–	–	–
Quillbot	✓	✓	–	✓	–	–
Gemini	–	✓	–	✓	–	–
Bard	–	✓	–	✓	–	–
Wordtune	–	✓	–	–	–	–
Research Rabbit	–	–	✓	✓	–	✓
Litmaps	–	–	✓	✓	–	✓
GPT-Zero	✓	–	–	–	–	–
iThenticate	✓	–	–	–	–	–
ChatPDF	–	–	✓	✓	–	✓
Covidence	–	–	✓	✓	–	✓
OpenEvidence	–	–	✓	✓	–	✓
Elicit	–	–	✓	✓	–	✓
Rayyan AI	–	–	✓	–	✓	✓
Scite AI	–	–	✓	–	–	–
CitationChaser	✓	–	✓	–	–	✓
Excel AI	–	–	–	–	✓	–

For example, researchers exploring intricate orthopedic topics—such as the biomechanics of joint replacements or the efficacy of minimally invasive surgical techniques—can use these tools to draft comprehensive sections, including background reviews, methodologies, and discussions [7]. An orthopedic resident studying advancements in anterior cruciate ligament (ACL) reconstruction could input findings into an AI tool, which would synthesize related literature, highlight research gaps, and structure the manuscript logically. This streamlines the writing process, allowing researchers to focus more on analysis and interpretation while alleviating the cognitive burden of managing large volumes of dense, academically complex information [9, 10].

Beyond drafting, AI tools are pivotal in refining manuscripts. Platforms such as Grammarly and Wordtune assist with grammar, syntax, and readability, ensuring that manuscripts meet the stringent standards of peer-reviewed journals. These tools provide essential support for non-native English speakers, offering accurate translations and enabling the creation of polished, globally competitive documents. By enhancing clarity and coherence, AI tools make it easier for researchers to communicate their findings effectively to a wide audience [9].

By automating routine tasks, enhancing the quality of writing, and providing critical support for non-native speakers, AI tools have become indispensable in academic writing. They empower orthopedic researchers and residents to focus on innovation and discovery while navigating the complexities of academic publishing with greater ease and efficiency [7, 10].

## Simplifying literature summarization and synthesis

Synthesizing information from large volumes of the literature is one of the most labor-intensive aspects of academic research, especially in fields like orthopedics, where evidence is continually evolving [10]. AI platforms such as Consensus and SciSpace have revolutionized this process by efficiently analyzing extensive datasets and condensing key findings into concise, organized summaries (Table 1). These tools enable researchers to process hundreds of studies in significantly less time, extracting insights categorized by variables such as methodology, patient outcomes, and surgical techniques [11].

For example, a researcher conducting a systematic review on the surgical management of musculoskeletal pathologies can leverage these tools to identify trends across a broad range of studies, compare outcomes of different surgical interventions, and organize findings into meaningful themes. This capability is particularly advantageous in rapidly evolving fields like orthopedics, where new technologies, techniques, and evidence emerge frequently. By cross-referencing insights with recent publications, AI ensures that content remains up-to-date, giving researchers a competitive edge in staying informed [9].

AI tools also enhance semantic understanding, addressing a common challenge in evidence synthesis: variability in terminology. For instance, studies may describe the same concept using different terms, such as “arthroplasty” versus “joint replacement.” AI algorithms can recognize these semantic overlaps, ensuring comprehensive retrieval of relevant studies and minimizing the risk of missing critical evidence. This semantic capability is essential for creating thorough and accurate literature reviews [9].

Beyond organizing and summarizing data, AI tools are expanding their role in evidence synthesis by addressing the Risk of Bias Assessment, a critical component of systematic reviews. These algorithms are being trained to evaluate study quality by identifying potential sources of bias, such as inadequate blinding, selective reporting, or incomplete data. Automating this process not only saves time but also enhances the rigor and reliability of systematic reviews, producing conclusions that are more robust and trustworthy [1, 9].

Moreover, AI-powered platforms facilitate deeper analyses by integrating metadata, such as sample sizes, geographic distribution of studies, and study designs, into their summaries. This helps researchers quickly identify patterns, such as regional variations in outcomes or disparities in study populations, providing insights that might otherwise be overlooked in manual reviews [10].

### Optimizing literature searches

Conducting comprehensive and accurate literature searches in databases such as PubMed, Scopus, and Embase is a cornerstone of academic research, but it can be labor-intensive and time-consuming [9]. AI-powered tools have significantly enhanced this process by optimizing search strategies and improving precision. For example, these tools can generate complex Boolean search strings tailored to specific research questions, incorporating synonyms, logical operators, and filters to refine search results. A query such as “arthroscopic repair AND rotator cuff tears NOT pediatric” can be dynamically expanded to include related terms, alternative spellings, and context-specific keywords, ensuring the search captures the most relevant studies.

By automating this optimization, AI reduces the burden of sifting through irrelevant or tangential results, enabling researchers to focus on analyzing high-quality evidence that directly supports their objectives [10]. This is particularly beneficial for systematic reviews and meta-analyses, where comprehensiveness and precision are paramount. For residents and early-career researchers, AI serves as a powerful tool to bridge the gap between their developing search skills and the expertise required for rigorous literature reviews [9].

AI tools also support researchers by suggesting related terms, highlighting potential gaps in search queries, and even ranking articles based on relevance to the research question. Additionally, these tools can assist in creating search strings that adhere to the requirements of specific databases, such as PubMed’s MeSH (Medical Subject Headings) terms, ensuring more accurate and comprehensive results. Advanced platforms can integrate search histories and refine strategies over time, tailoring recommendations as researchers iterate their queries [12].

It is important to note, however, that tools like ChatGPT do not have direct access to subscription-protected databases such as PubMed, Scopus, or Embase due to copyright and access restrictions. Instead, ChatGPT relies on its pre-trained knowledge (up to October 2023) and cannot retrieve or analyze live database content [10]. Researchers must use dedicated AI-driven platforms that are explicitly designed for database integration to achieve direct interaction with these resources.

Despite this limitation, AI tools remain invaluable for generating search strategies, offering insights into the structure of effective queries, and providing general guidance on relevant topics. When combined with access to specialized platforms, AI helps researchers achieve more efficient, accurate, and comprehensive literature searches, ultimately improving the quality of their evidence-based research [11].

AI-powered tools such as Litmaps and Research Rabbit are transforming how researchers summarize and synthesize academic literature, making the exploration of complex research landscapes more intuitive and efficient. Litmaps allow researchers to visualize the evolution of ideas through interactive citation maps. By plotting connections between studies and tracking how research progresses over time, this tool highlights key papers, influential authors, and emerging trends in a field. Litmaps also automatically updates users about new citations relevant to their research, removing the need for manual monitoring and ensuring that researchers remain informed about the latest developments (Table 1).

Research Rabbit, on the other hand, provides a dynamic and exploratory approach to literature discovery. Acting as a virtual research assistant, it suggests related articles based on a given paper, topic, or author. Its unique ability to map citation networks and highlight connections between authors and co-authors reveals patterns and relationships that are often overlooked in traditional database searches. This feature not only enhances the depth of literature reviews but also uncovers collaborative opportunities and emerging areas of study (Table 1).

By integrating features such as citation tracking, network visualization, and intelligent recommendations, these tools streamline the exploration and organization of academic knowledge. They free researchers from repetitive tasks, enabling them to concentrate on deeper analysis and innovative thinking. Whether used to identify gaps in existing research, monitor trends, or organize findings for systematic reviews, Litmaps and Research Rabbit exemplify the power of AI in elevating the efficiency and effectiveness of scholarly work (Table 1).

### Enhancing citation management and formatting

Efficient citation management is a cornerstone of academic writing, and AI-powered tools such as Zotero, Mendeley, and EndNote are revolutionizing this process [13]. These platforms automate the storage, organization, and formatting of references, allowing researchers to create and maintain personalized libraries for their projects. Users can tag references, add annotations, and categorize sources, enabling quick retrieval and seamless integration into manuscripts. With just a few clicks, bibliographies can be generated in various styles, such as APA, AMA, Chicago, or Vancouver, meeting the specific requirements of different journals or academic fields [13].

One of the most valuable features of these tools is their adaptability. For instance, if a manuscript needs to be reformatted to meet the guidelines of a different journal, these platforms can instantly reformat all citations and the bibliography, saving significant time and effort. This functionality is especially beneficial for researchers submitting to multiple journals with distinct formatting requirements [9].

Collaboration is another area where these tools excel. Zotero and Mendeley, for example, allow co-authors to share and edit reference libraries in real-time. This feature is particularly useful in multi-center studies, where researchers from various institutions need to coordinate bibliographic data. Shared libraries ensure that all team members have access to the latest citations, fostering better collaboration and reducing inconsistencies in reference management [13].

AI further enhances citation management by introducing intelligent search capabilities. Tools integrated with AI can analyze a manuscript’s content and suggest relevant articles for citation, ensuring that references are not only accurate but also contextually appropriate. By automating the search and suggestion process, these tools help researchers identify the most pertinent literature, improving the depth and quality of their work. This is particularly advantageous for researchers working under tight deadlines or handling large volumes of the literature [9].

In addition, AI tools can identify and flag missing citations, ensuring that all referenced materials are properly cited in the bibliography. They can even verify citation accuracy by cross-checking with online databases, reducing errors, and ensuring compliance with academic standards. Some advanced tools also assist in identifying highly cited or seminal works in a specific field, helping researchers position their work within the broader academic landscape [9].

### Plagiarism detection with AI

AI-powered plagiarism detection tools play a critical role in maintaining the integrity and originality of academic writing, especially in an era where Generative Artificial Intelligence (GAI) platforms such as ChatGPT, Gemini, and QuillBot have transformed how knowledge is sourced and composed. Tools such as GPT-Zero, iThenticate, and Grammarly use advanced algorithms to compare manuscripts against extensive databases of published articles, websites, and academic content (Table 1). They identify potential plagiarism by highlighting text overlaps, paraphrased content, or improperly cited material, enabling researchers to address these issues before submission [14]. However, the increasing sophistication of AI-generated text is making it progressively more challenging for traditional plagiarism detection tools to distinguish between original and AI-assisted content [14].

For orthopedic researchers, these tools are particularly valuable in ensuring that manuscripts adhere to the ethical standards required by peer-reviewed journals. By providing detailed similarity reports, they help authors refine their writing, properly attribute ideas, and avoid inadvertent plagiarism—a common challenge when synthesizing large volumes of the literature. Moreover, AI-powered plagiarism detectors can suggest citation improvements and optimize reference management, further enhancing the credibility and professionalism of research [14].

Despite these advantages, recent studies highlight the growing difficulty of identifying AI-generated content accurately. As tools such as ChatGPT and QuillBot evolve to paraphrase and summarize text seamlessly, educators and researchers increasingly question the reliability of current plagiarism detection algorithms. This ambiguity underscores the need for plagiarism detectors to adapt by incorporating semantic understanding and advanced AI techniques to address the complexities of identifying “machine-assisted” writing [9, 14, 15].

Additionally, in collaborative research environments or when working with large datasets, AI plagiarism detectors ensure consistency and accountability. They facilitate trust among collaborators and publishers by standardizing the integrity of academic submissions. While these tools automate a critical aspect of academic integrity, researchers must remain vigilant, as over-reliance on similarity scores without deeper contextual evaluation may inadvertently support rather than prevent plagiarism [10].

In this rapidly changing landscape, AI plagiarism detection tools are no longer just safeguards but integral components of academic writing workflows. They not only help researchers maintain ethical standards but also highlight the evolving challenges in defining originality in a world increasingly shaped by generative AI. As such, the orthopedic research community—and academia at large—must strive to refine how these tools are utilized to ensure both transparency and trustworthiness in scientific communication [12].

### Statistical analyses with AI

AI tools are reshaping the landscape of statistical analysis by providing intuitive, accessible solutions for both descriptive and inferential statistics. Generative AI platforms enable researchers to perform tests such as t-tests, ANOVA, chi-square tests, and regression analyses with remarkable ease, often without requiring advanced programming skills [9, 10]. These tools go beyond executing calculations by offering step-by-step guidance, such as explaining test assumptions, interpreting p-values, and calculating effect sizes, making complex statistical concepts more approachable for researchers.

AI tools also facilitate more complex analyses, such as multivariate regression, survival analyses, and structural equation modeling, by automating much of the coding and computational process. This is particularly valuable for orthopedic research, where large datasets are frequently used to examine clinical outcomes, patient recovery trajectories, and risk factors for complications [9].

Importantly, AI-generated statistical results must be validated by biostatisticians or experienced analysts to ensure accuracy and relevance. While these tools are powerful, they rely on user-provided data and assumptions, which can introduce errors if not carefully vetted. A collaborative approach, where AI simplifies the execution of statistical tests and biostatisticians verify the results, ensures that the analyses are both technically sound and contextually appropriate to the research question [10].

In addition to facilitating analyses, AI can generate code for statistical tests in languages such as R or Python, enabling reproducibility and sharing among research teams [16]. For instance, an orthopedic resident can input a dataset and request a script for a multivariate analysis, which the AI generates with clear documentation, allowing others to replicate or modify the analysis with ease. This capability promotes transparency and collaboration in research.

### Interpreting graphical data

AI is transforming the interpretation of statistical graphics in orthopedics by automating the analysis of complex visual data, such as Kaplan–Meier survival curves, forest plots, ROC curves, and scatter plots. These tools not only extract critical information efficiently but also present it in a more accessible format, making it easier for orthopedic researchers and clinicians to identify trends, evaluate outcomes, and draw meaningful conclusions from dense datasets [9].

Kaplan–Meier curves, for example, are essential for understanding survival probabilities over time, often requiring careful interpretation of censored data and group comparisons. AI systems can automatically detect inflection points, calculate hazard ratios, and flag statistically significant differences between groups, reducing the cognitive load on researchers. Similarly, forest plots used in meta-analyses are simplified with AI assistance, which highlights pooled effect sizes, confidence intervals, and study heterogeneity, enabling rapid assessment of overall trends [10].

By automating the interpretation of statistical graphics, AI demystifies complex visuals and equips residents with the skills to critically evaluate findings from published studies. This not only accelerates their learning but also boosts their confidence in applying evidence-based practices in clinical settings.

Moreover, the integration of AI for interpreting statistical graphics reduces the risk of human error, which can arise from misreading or miscalculating data points. This reliability is especially crucial in orthopedics, where accurate interpretation of outcomes directly impacts surgical planning, rehabilitation protocols, and patient care strategies [2]. By combining speed, accuracy, and accessibility, AI not only enhances the quality of research but also supports clinicians in making informed, data-driven decisions that improve patient outcomes.

### Generating statistical visualizations

AI platforms integrated with programming environments such as Python and R are transforming the way researchers create and utilize statistical visualizations [16]. These tools enable the effortless generation of advanced graphical outputs, such as subgroup analyses and sensitivity plots for systematic reviews, as well as comparative bar charts, scatterplots, and histograms for illustrating clinical outcomes. These visualizations are not only vital for presenting data clearly but also for uncovering trends, relationships, and insights that drive evidence-based decision-making [9].

A key advantage of these AI tools is their ability to generate customized code for platforms such as R or Python. Researchers can input statistical data or specify the type of visualization they need, and the AI generates the corresponding script to create the desired graphic. This process greatly streamlines the workflow, eliminating the need for manual coding and reducing the learning curve for researchers with limited programming experience [10]. For instance, an orthopedic researcher could request a Kaplan–Meier survival plot or a forest plot for meta-analysis, and the AI would provide precise, ready-to-use code tailored to the dataset.

Moreover, these tools enhance accessibility by democratizing the creation of high-quality visualizations. They empower researchers from diverse backgrounds, including those in low-resource settings or with limited technical expertise, to produce professional-grade graphics that meet publication standards. This capability not only saves time but also allows researchers to focus more on interpreting data and less on technical execution.

Additionally, these AI-driven solutions are valuable for fostering collaboration. By standardizing the visualization process and simplifying the sharing of reproducible code, they ensure that teams can efficiently communicate findings and work seamlessly across disciplines. Whether used for manuscript preparation, presentations, or interdisciplinary collaboration, AI-powered visualization tools are becoming indispensable in modern research, enhancing both efficiency and the quality of scientific communication.

### Ethical considerations and challenges

The integration of AI into orthopedic research holds immense potential but also introduces significant ethical and practical challenges [10, 12]. A key concern is the quality and reliability of AI-generated information, as biases or inaccuracies in training datasets can compromise the validity of outputs. This issue is compounded by the “black box” nature of many AI algorithms, which can erode trust among clinicians and researchers when critical decisions, such as patient care or surgical planning, are at stake [17, 18].

Ethical concerns, such as data privacy and algorithmic transparency, further complicate AI’s adoption. Protecting patient privacy requires robust anonymization strategies and strict compliance with regulations such as GDPR and HIPAA. Additionally, ensuring that AI training datasets are diverse is essential to minimize bias and enable equitable performance across different populations [12].

To address these challenges, orthopedic residents and researchers must be educated on the ethical implications and limitations of AI tools. This training is critical to empowering them to evaluate AI-generated insights critically and use these technologies responsibly [19]. Successful integration also demands collaboration among clinicians, data scientists, and regulatory bodies to validate AI models, establish transparent guidelines, and foster trust in these systems [12].

As AI continues to advance across all professional fields, orthopedic surgeons must take advantage of the benefits these tools offer while also addressing the challenges they pose to daily practice [12]. Not only are these technologies valuable as research assistants, but they also provide numerous advantages in the field of orthopedic surgery [3]. To remain at the forefront of innovation, it is essential to proactively implement AI tools, ensuring that their potential to improve both clinical practice and research is fully realized. By doing so, orthopedic professionals can maximize the impact of AI, improving efficiency, accuracy, and ultimately patient care.

### AI literacy in residency programs

The successful integration of AI into orthopedic research and practice hinges on building AI literacy within residency programs. To fully harness the potential of these technologies, orthopedic residents must receive comprehensive training in both the ethical and practical aspects of AI. This includes developing the ability to critically assess the validity, reliability, and biases of AI-generated outputs, as well as understanding the limitations and appropriate contexts for their use in clinical decision-making and research. Beyond technical proficiency, residents must also be educated on the ethical implications of AI, such as issues related to patient privacy, algorithmic bias, and the potential for AI to perpetuate healthcare disparities. Furthermore, interdisciplinary collaboration with data scientists and AI specialists should be encouraged, fostering a research culture that integrates AI responsibly and innovatively. Incorporating AI literacy into residency programs will empower the next generation of orthopedic surgeons to use AI tools thoughtfully, ensuring that these technologies enhance, rather than replace, clinical judgment. Ultimately, a well-rounded understanding of AI will enable future orthopedic surgeons to optimize patient outcomes, drive innovation in research, and contribute to the responsible evolution of AI in medicine.

### Reducing burnout and promoting equity in research

AI tools are transforming research by democratizing access to high-quality resources, particularly benefiting researchers in low-resource settings or regions where English is not the primary language. By automating repetitive and time-consuming tasks, these tools save significant time and effort, allowing researchers to maintain productivity while reducing the risk of burnout [20].

For orthopedic residents balancing the rigorous demands of clinical practice and research, these advancements are transformative. AI enables them to focus on the intellectual and creative aspects of their work—such as developing hypotheses and designing studies—rather than being bogged down by administrative tasks [21].

Furthermore, in evidence synthesis, AI significantly improves the efficiency and accuracy of different types of research such as systematic reviews and meta-analyses, streamlining processes that traditionally required a great deal of manual effort [21]. By reducing burnout and promoting equity in research, AI serves as a critical tool in modern orthopedic research.

## Conclusion

Artificial intelligence is transforming orthopedic research, enabling researchers to automate writing, synthesize evidence, and analyze complex datasets with unprecedented efficiency. For orthopedic residents, AI promotes independence and enhances learning, reducing reliance on external expertise while expanding research capabilities. By addressing ethical challenges and integrating AI into educational frameworks, the orthopedic community can fully harness its transformative potential to advance research, improve patient care, and shape the future of evidence-based practice.

## Data Availability

No datasets were generated or analyzed during the current study.
